# Supplementary material for: QSPR Modeling and Experimental Determination of the Antioxidant Activity of Some Polycyclic Compounds in the Radical-Chain Oxidation Reaction of Organic Substrates
Source: Molecules. 2022 Oct 2;27(19):6511. doi: 10.3390/molecules27196511 (PMC9572093; doi:10.3390/molecules27196511)
Supplement: Supplementary file 1 [file molecules-27-06511-s001.zip › molecules-1906655-supplementary.pdf]

SupplementaryMaterial

1. Kinetic Classification of Antioxidants Depending on The Deactivation Mode Of Oxidation

1. Antioxidants terminating the chains by their reactions with peroxyl radicals (phenols, naphthols, hydroquinones, aromatic amines, aminophenols, diamines) resulting in the formation of radical intermediates with low activity.

2. Antioxidants terminating the chains by their reactions with alkyl radicals (quinones, nitrones, iminoquinones, methylenequinones, stable nitroxyl radicals, and nitro compounds). Such antioxidants are efficient at very low concentrations of dioxygen and in solid polymers).

3. Antioxidants decomposing hydroperoxide (sulfides, phosphites, arsenites, thiophosphates, carbamates, and some metal complexes) without forming free radicals. Reactions with hydroperoxides can be either stoichiometric (e.g., with sulfides and phosphites) or catalytic (e.g., chelate metal complexes).

4. Metal-deactivating antioxidants (diamines, hydroxy acids, and other bifunctional compounds) interacting with metal ions and forming the complexes inactive towards hydroperoxides.

5. Cyclic chain termination by antioxidants (aromatic amines, nitroxyl radicals, and variable-valence metal compounds).

6. Inhibitors with combined action. Such a mechanism is realized when (1) the inhibitor molecule has two and more functional groups undergoing their own reaction; and (2) the original inhibitor and its products of its transformation possess the inhibitory activities through different inhibition modes (e.g., the phenolic group of phenol sulfide reacts with peroxyl radical whereas its sulfide group is reactive towards hydroperoxide).

7. Synergetic inhibition is implemented when two inhibitors mutually enhance their inhibitory effects (e.g., in the case of 'phenol + sulfide' mixtures, in which phenol reacts with the peroxyl radical and sulfide reduces the degenerate chain branching by non-radical decomposition of hydroperoxide).

In the aspect above, a quantitative study of the antioxidant properties of natural and synthetic substances in various model systems is an important task. Assessing the antioxidant activity of individual substances and compositions may be performed with various

physicochemical and biochemical methods is used [7]. This can be done according to their influence on the oxygen absorption (lipid peroxidation, aromatic hydrocarbons, secondary and tertiary alcohols, oxidation of crocin, chemiluminescence with luminol, oxidation of R-phycoerythrin, sensitivity of erythrocytes to hemolysis, recovery of the activity of iron ions, lipid peroxides). Some authors measure the antioxidant activity of enzymes, e.g., ascorbate-peroxidase, glutathione reductase, dehydroascorbate reductase and mono-dehydroascorbate reductase. Herewith, in some cases, the antioxidant status of the organism correlates with the intensity of the pathology, e.g., the growth of malignant tumor cells MK-1.

Despite the diverse photometric, chromatographic and electrochemical methods, a study of the antioxidant activity (AOA) of individual compounds usually starts from the methods of chemical kinetics. In these methods, AOA compounds are involved to the model reactions such as oxidation of aliphatic and alkyl-aromatic hydrocarbons, fatty acid esters. Here, the antioxidant efficacy is estimated by the duration and depth of the inhibition of oxidation of model substrates. The main advantages of the kinetic methods for the AOA assessment are their accessibility, possibility of standardization of the substrates and the oxidation regime. The mentioned features are necessary for the reproducibility of the results [2]. A strict kinetic description of the oxidation processes and measurement of the corresponding rate constants of the elementary stages are the main advantages of this approach. However, it should be noted that the kinetic methods are non-selective to a specific antioxidant when studying the antioxidant properties of extractive compositions and mixtures of biologically active substances with a pronounced antioxidant effect [7].

2. Parameters for Assessing the Descriptive and Predictive Potential of QSAR Models

Table S1. The equations for assessing the descriptive and predictive potentials of the QSAR models based on the R^2 and MAE metrics

Comment	Equation of the criterion	
Parameters for assessing the descriptive and predictive potential of QSAR models using internal cross-validation techniques		
Determination coefficient (Coefficient of multiple determination R^2) is the	$$R^2 = 1 - \frac{\sum_{i=1}^{N_{TRi}} (y_i^{pred} - y_i^{obs})^2}{\sum_{i=1}^{N_{TRi}} (y_i^{obs} - \overline{y^{obs}})^2} = 1 - \frac{RSS}{TSS}$$	(1)

determination coefficient of the calculated using the experimental and the predicted data of the training set	$R^2 = \left(\dfrac{\sum_{i=1}^{N_{TRi}} (y_i^{obs} - \overline{y^{obs}})(y_i^{pred} - \overline{y^{pred}})}{\sqrt{\sum_{i=1}^{N_{TRi}} (y_i^{obs} - \overline{y^{obs}})^2 \times \sum_{i=1}^{N_{TRi}} (y_i^{pred} - \overline{y^{pred}})^2}} \right)^2$	
R_0^2 and $R_0'^2$ are respectively the determination coefficients of the calculated using the experimental and the predicted data of the training set, forcing respectively the origin of the axis	$R_0^2 = 1 - \dfrac{\sum_{i=1}^{N_{TRi}} (y_i^{pred} - k \bullet y_i^{pred})^2}{\sum_{i=1}^{N_{TRi}} (y_i^{pred} - \overline{y^{pred}})^2}$ $R_0'^2 = 1 - \dfrac{\sum_{i=1}^{N_{TRi}} (y_i^{obs} - k' \bullet y_i^{obs})^2}{\sum_{i=1}^{N_{TRi}} (y_i^{obs} - \overline{y^{obs}})^2}$ $k = \dfrac{\sum_{i=1}^{N_{TRi}} (y_i^{obs} \text{晊}_i^{pred})}{\sum_{i=1}^{N_{TRi}} (y_i^{pred})^2}$; $k' = \dfrac{\sum_{i=1}^{N_{TRi}} (y_i^{obs} \text{晊}_i^{pred})}{\sum_{i=1}^{N_{TRi}} (y_i^{pred})^2}$	(2)
$R^2{}_m$ is determination coefficient of the regression function, calculated using the experimental values on the ordinate axis, $R'^2{}_m$ using them on the abscissa	$R_m^2 = R_{TRi}^2 (1 - \sqrt{R_{TRi}^2 - R_{0/TRi}^2}) > 0.5$ $\Delta R_m^2 = \lfloor R_m^2 - R_m'^2 \rfloor < 0.2$ $\overline{R_m^2} = \dfrac{R_m^2 + R_m'^2}{2}$	(3)
Determination coefficient by internal cross-validation	$Q^2 = Q^2_{20\%(n=20)} = 1 - \dfrac{\sum_{i=1}^{N_{TRi}} (y_{i/i}^{pred} - y_i^{obs})^2}{\sum_{i=1}^{N_{TRi}} (y_i^{obs} - \overline{y^{obs}})^2} = 1 - \dfrac{PRESS}{TSS}$	(4)
Standart deviation	$S.D. = \sqrt{\dfrac{\sum_{i=1}^{N_{TRi}} (y_i^{obs} - y_i^{pred})^2}{N_{TRi} - V - 1}} = \sqrt{\dfrac{RSS}{N_{TRi} - V - 1}}$	(5)
Root Mean Square Error in prediction activity for training set	$RMSE = \sqrt{\dfrac{\sum_{i=1}^{N_{TRi}} (y_i^{obs} - y_i^{pred})^2}{N_{TRi}}} = \sqrt{\dfrac{RSS}{N_{TRi}}}$	(6)
Variance ratio (F)	$F = \dfrac{\sum_{i=1}^{N_{TRi}} (y_i^{pred} - \overline{y^{obs}})^2}{\sum_{i=1}^{N_{TRi}} (y_i^{obs} - y_i^{pred})^2} \times \dfrac{N_{TRi} - V - 1}{V}$	(7)
Parameters of assessing the descriptive and predictive abilities of QSAR models within the external cross-validation techniques		

R_0^2 and $R_0'^2$ are calculated forcing the regression line to pass through the origin, k and k' are the slope of the regression lines	$R_0^2 = 1 - \frac{\sum_{i=1}^{N_{TSi}} (y_i^{pred} - k \bullet y_i^{pred})^2}{\sum_{i=1}^{N_{TSi}} (y_i^{pred} - \overline{y^{pred}})^2}$ $R_0'^2 = 1 - \frac{\sum_{i=1}^{N_{TSi}} (y_i^{obs} - k' \bullet y_i^{obs})^2}{\sum_{i=1}^{N_{TSi}} (y_i^{obs} - \overline{y^{obs}})^2}$ $k = \frac{\sum_{i=1}^{N_{TSi}} (y_i^{obs} \text{瞠} y_i^{pred})}{\sum_{i=1}^{N_{TSi}} (y_i^{pred})^2}$; $k' = \frac{\sum_{i=1}^{N_{TSi}} (y_i^{obs} \text{瞠} y_i^{pred})}{\sum_{i=1}^{N_{TSi}} (y_i^{pred})^2}$	(8)
Correlation coefficient between observed and predicted activities	$R_{TSi}^2 = 1 - \frac{\sum_{i=1}^{N_{TSi}} (y_i^{pred} - y_i^{obs})^2}{\sum_{i=1}^{N_{TSi}} (y_i^{obs} - \overline{y^{obs}})^2} = 1 - \frac{PRESS}{TSS}$ $R_{TSi}^2 = \left(\frac{\sum_{i=1}^{N_{TSi}} (y_i^{obs} - \overline{y^{obs}})(y_i^{pred} - \overline{y^{pred}})}{\sqrt{\sum_{i=1}^{N_{TSi}} (y_i^{obs} - \overline{y^{obs}})^2 \times \sum_{i=1}^{N_{TSi}} (y_i^{pred} - \overline{y^{pred}})^2}} \right)^2$	(9)
Determination coefficients calculated for compounds of test set TS_i, taking into account lgk_7 for compounds of the training set and average lgk_7 for compound of the test set	$Q_{F1}^2 = 1 - \frac{\sum_{i=1}^{N_{TSi}} (y_i^{pred} - y_i^{obs})^2}{\sum_{i=1}^{N_{TSi}} (y_i^{obs} - \overline{y_{i/TRi}^{obs}})^2} = 1 - \frac{PRESS}{TSS_{test}(\overline{y_{i/TRi}^{obs}})}$	(10)
	$Q_{F2}^2 = 1 - \frac{\sum_{i=1}^{N_{TSi}} (y_i^{pred} - y_i^{obs})^2}{\sum_{i=1}^{N_{TSi}} (y_i^{obs} - \overline{y_{i/TSi}^{obs}})^2} = 1 - \frac{PRESS}{TSS_{test}(\overline{y_{i/TSi}^{obs}})} = R_{TSi}^2$	(11)
Concordance Correlation Coefficient (CCC)	$CCC = \frac{2\sum_{i=1}^{N_{TSi}} (y_i^{obs} - \overline{y^{obs}})(y_i^{pred} - \overline{y^{pred}})}{\sum_{i=1}^{N_{TSi}} (y_i^{obs} - \overline{y^{obs}})^2 + \sum_{i=1}^{N_{TSi}} (y_i^{pred} - \overline{y^{pred}})^2 + N_{TSi}(\overline{y^{obs}} - \overline{y^{pred}})}$	(12)
R^2_m is determination coefficient of the regression function, calculated using the experimental values on the ordinate axis, R'^2_m using	$R_m^2 = R_{TSi}^2 (1 - \sqrt{R_{TSi}^2 - R_{0TSi}^2} > 0.5$ $\Delta R_m^2 = \lfloor R_m^2 - R_m'^2 \rfloor < 0.2$ $\overline{R_m^2} = \frac{R_m^2 + R_m'^2}{2}$	(13)

them on the abscissa		
Root Mean Square Error in prediction activity for test set	$$RMSEP = \sqrt{\frac{\sum_{i=1}^{N_{TSi}} (y_i^{obs} - y_i^{pred})^2}{N_{TSi}}} = \sqrt{\frac{RSS}{N_{TSi}}}$$	(14)
Mean Absolute Error	$$MAE = \frac{\sum_{i=1}^{N_{TSi}} \left| y_i^{obs} - y_i^{pred} \right|}{N_{TSi}}$$	(15)

where

TRi is the training set, TSi is the test set,

N_{TRi} and N_{TSi} are total number of objects in the training set and test set respectively;

y_i^{obs} are experimental data values, y_i^{pred} are predicted data values;

$\overline{y^{obs}}$ are average of the experimental data values;

$\overline{y^{pred}}$ are average of the predicted data values;

RSS is residual sum of squares;

PRESS is the sum of the squares of the prediction errors (predictive sum of squares);

TSS is the total sum of squares (is sum of squared deviations from the data set mean);

$TSS_{test}(\overline{y_{i/train}^{obs}})$ and $TSS_{test}(\overline{y_{i/test}^{obs}})$ are the total sum of squares of the external set calculated using the training set mean and external set mean, respectively.

3. Briefdescription of the Program Gusar 2019

3.1. Calculation of Structural Descriptors

Here is a description of the GUSAR program necessary to understand the text of the article.

A detailed description of the ideology of calculating descriptors and constructing QSAR models using this program is given in the articles listed in the list of references and in the site http://www.pharmaexpert.ru [55].

In the GUSAR2019 program, the description of the structure and the calculation of the regression coefficients for the further construction of QSAR models is based on the use of two types of substructural descriptors of atomic neighborhoods: MNA (Multilevel Neighborhoods of Atoms) and QNA (Quantitative Neighborhoods of Atoms) [59,60]. They are automatically deduced from the matrices of molecular connectivity, standard ionization

potentials (IP) and electron affinities (EA). The QNA descriptors are defined by two functions, P and Q. The P and Q values for each atom *i* are calculated using the following formulae [60]:

$$P_i = B_i \sum_k \left(\exp\left(-\frac{1}{2} C \right) \right)_{ik} B_k \quad (1)$$

$$Q_i = B_i \sum_k \left(\exp\left(-\frac{1}{2} C \right) \right)_{ik} B_k A_k \quad (2)$$

$$A_k = \frac{1}{2}(IP_k + EA_k), \; B_k = (IP_k - EA_k)^{-1/2} \quad (3)$$

where *k* is the remaining atoms in the molecule, IP is the first ionization potential, EA is the electron affinity for each atom (in eV), and C is the connectivity matrix for the molecule as a whole [87]. The standard values IP and EA of atoms in a molecule were collected from the literature. A detailed description of QNA descriptors is represented in [59].

Thus, the QNA descriptors are calculated taking into account the relationships between all atoms of the structure. These values describe each atom of the molecule but, at the same time, depend on the structure of the molecule as a whole [59,88]. The QNA values are the basic information for calculating the Chebyshev 2D polynomials. It is important to note that in the final QSAR models, the independent variables include the mean values of the individual two-dimensional Chebyshev polynomials from the P and Q values calculated for all atoms in the molecule. Thus, the regression equations constructed in the GUSAR2019 program take into account both the specificity and physicochemical properties of each atom entering the training set [2,7]. However, QNA descriptors cannot be physically interpreted due to the peculiarities of their calculation. In this regard, they are not explicitly displayed under calculations.

The MNA descriptors are computed using the PASS algorithm (Prediction of Activity Spectra for Substances) [17,60], which predicts approximately 6,400 "biological activities" with an accuracy threshold of an average prediction of at least 95%. These descriptors are generated based on the structural formulae of chemical compounds without using any pre-compiled list of structural fragments [11,17,60,88]. The authors of the GUSAR2019 program report that "MNA-descriptors are based on the molecular structure representation, which includes hydrogens according to the valences and partial charges of other atoms and does not specify the types of bonds." They are generated as "a recursively defined sequence:

• zero-level MNA descriptor for each atom is the mark A of the atom itself;

• any next-level MNA descriptor for the atom is the substructure notation A ($D_1D_2 \ldots D_i \ldots$), where Di is the previous-level MNA descriptor for i–th immediate neighbor of the atom A.

The neighbor descriptors $D_1D_2 \ldots D_i \ldots$ are arranged in a unique manner. This may be, for example, a lexicographic sequence. MNA descriptors are generated using an iterative procedure, which results in the formation of structural descriptors that include the first, second, etc. neighborhoods of each atom. The label contains not only information about the type of atom, but also additional information about its belonging to a cyclic or acyclic system, etc. For example, an atom that does not enter a ring is marked with a "—".

Based on the MNA descriptors using B-statistics, calculated in the PASS program, the biological activity spectrum of a chemical compound is predicted [17,61–66,87,88].

The output of the PASS program is the probabilities of the activity (Pa) and of inactivity (Pi) of each prognostic result. The difference between these two values (Pa–Pi) for a randomly selected subset of predicted activities is used as independent variables for regression analysis in GUSAR. GUSAR2019 incorporates a PASS version that pedicts 4130 types of biological activity. The developers of the GUSAR2019 program report that the list of predictable biological activities currently includes 501 pharmacotherapeutic effects, 3295 mechanisms of action, 57 adverse and toxic effects, 199 metabolic terms, 49 transporter proteins and 29 activities related to gene expression [88]. The average accuracy of a reliable prediction of biological activity, calculated by leave-one-out cross-validation procedure is approximately 95% [71]. However, the regression equation constructed based on the MNA descriptors reveals the specificity of the action of the compound but does not explicitly reflects the physicochemical parameters of chemical compounds [88].

In addition, the GUSAR2019 program calculates the QSAR descriptors of an entire molecule such as topological length, topological volume, lipophilicity, and physicochemical descriptors (numbers of positive and negative charges, number of donors and acceptors of the hydrogen bond, number of aromatic atoms, molecular weight and number of halogen atoms) [17,60].

The authors of the GUSAR2019 program report that "in GUSAR, the scale of QNA- and PASS-based descriptors ranges from −1 to 1. Therefore, no additional normalization is required for these types of descriptors. Only whole-molecule descriptors are normalized using a standard Z-score normalization procedure" [17].

It should be noted that the program is able to construct QSAR models both relying solely on one of these types of descriptors, and on their combination in terms of the consensus

approach [61-66]. At the same time, based on the consensus approach methodology, models for quantitative prediction of biological activity for these descriptors are calculated independently of each other. The examples of the sample QSAR GUSAR models for predicting the toxic effects of chemical compounds are available free *via* the link http://www.way2drug.com/GUSAR.

However, it noteworthy that the features of the QNA and MNA calculations retain these descriptors without unambiguous physical interpretation. For this reason, in the commercial and academic versions of the GUSAR2019 program for broad use, the regression equations are not displayed.

3.2. Selection of the Descriptors When Consrtucting Qsar Models

In GUSAR2019, three approaches are used when selecting the optimal number of descriptors for constructing (Q)SAR-models:

1) self-consistent regression method (SCR) [57-59];

2) method of radial basis functions (RBF) [60];

3) method based on the combination of SCR and RBF [60].

The SCR and RBF-SCR methods are the most preferable. The SCR method is correctly applied to modeling compounds with a rather high degree of similarity. The other two methods of selecting the optimal number of descriptors show good results when modeling structurally dissimilar compounds.

It was previously shown [11,15,60–66] that self-consistent regression (SCR) can be successfully applied to various QSAR problems. The SCR method is resistant to noise in the data and allows deleting the variables that poorly describe the target value. This is a regularized method of the least squares. Independent parameters a are calculated in this method according to the equation (4) [54]:

$$a = \mathrm{ArgMin}\left[\sum_{i=1}^{n} y_i - \sum_{k=0}^{m} x_{ik} a_k)^2 + \sum_{k=1}^{m} v_k a_k^2\right] \quad (4)$$

where a is the regression coefficient, n is the number of objects, y_i is the response value of the i-th object, m is the number of independent variables, x_{ik} is the value of the k-th independent variable of the i-th object, a_k is the k-th value of the regression coefficients, and v_k is the k-th value of the regularization parameters. Equation (4) hasthefollowingsolution:

$$a = TX^T y, \quad T = (X^T X + V)^{-1}$$

where X^T is the transposed regression matrix X, and V is the diagonal matrix of the regularization parameters. The regression coefficients obtained from the SCR reflect the

contribution of each particular descriptor (variable) to the final equation. The higher the absolute value of the coefficient, the greater its contribution. Thus, the regression coefficients obtained after the SCR can be used to weight the descriptors (variables) depending on their importance.

The second method used implemented in the GUSAR2019 program for selecting the optimal number of descriptors is the interpolation method for radial basis functions RBF [60]. The authors of the GUSAR2019 program reports [60] that, unlike the RBF network, this method uses each input variable as a center of gravity. The learning process is performed on all input variables of the training set. As can be seen from equation (5), the approximating function y(x) in the case of the RBF interpolation is represented as the sum of *N* radial basis functions, each of which is related to another center x_i and weighted by the corresponding coefficient w_i.

$$y(x) = \sum_{i=1}^{N} w_i \varphi(\|x - x_i\|) = \Phi w \qquad (5)$$

If the points x_i are different then the interpolation matrix Φ in the above equation is nonsingular. The weights w are calculated as:

$$w = \Phi^{-1} y \qquad (6)$$

Assessing the weights is based on the simple least squares method [60].

The RBF-SCR method is the third tool of the GUSAR2019 program for selecting the optimal number of descriptors. It has a 3-step algorithm:

1) selecting descriptors using the SCR method;
2) calculating the radial basis functions using the weighted coefficient of SCR as a criterion of similarity;
3) calculating the weighting coefficients RBF by the least squares.

The RBF-SCR method can be expressed as [60]:

$$y(x) = \sum_{i=1}^{N} w_i \varphi(\|ax - a_i x_i\|) = \Phi w \qquad (7)$$

where a is taken from equation (4). Weights a_i are a new elements as compared to equation (5).

The RBF and RBF-SCR interpolation is based on a linear radial basis function that allows modeling a variety of training sets with a high level of dissimilarity between the objects.

Additionally, the GUSAR program allows visualizing the contribution of each atom into the predicted value [60–66]. This capability is implemented in the QSAR models based

on the QNA descriptors and, accordingly, in the consensus combination of the QSAR models designed in different modes. It opens opportunities to identify "strong" and "weak" points in the biologically active molecules and, consequently, to rationalize the conclusions about the replacement of certain fragments upon molecular design directed to enhancing/weakening the target property.

3.3. Constructing of the Qsar Models

The QSAR models were designed in the GUSAR2019 program as follows. To describe the structures of compounds within the program, two types of atom-centered descriptors were used, *viz.*substructural MNA, electrotopological QNA, and, additionally, three descriptors of the whole molecule (topological length, topological volume, and lipophilicity).

The optimal set of the descriptors for constructing particular regression equations was automatically selected by the self-consistent regression [60] and sliding control procedures [60–66]. The GUSAR2019 program allows constructing any single QSAR models and consensus models based on them. In this study, we use the consensus approach to construct the QSAR models. This allows reducing the variability of the predictions. Consensus models were designed in GUSAR2019 automatically based on the principle of common similarity of particular regression dependencies [60-66].

The final predicted values for lgk_7 were calculated using a weighted average of the predictions from several selected QSAR models. Each model is based on a different set of QNA and MNA descriptors. Its predictions for each compound are weighted according to the similarity value as calculated during the applicability domain assessment. Note that each of these partial models involved by the consensus model was made independently based on either QNA or MNA descriptors. As a result, 9 consensus QSAR models were designed. These models included 140 partial models. However, not all of them had acceptable statistical parameters. To select the most predictive models, a 20-fold crosscheck was performed for each model. These models have the R^2 values exceed 0.6 (from the cross-validation procedure after the randomized rejection of 20% of the training set). Each of the final consensus models M1–M2, M4–M5, M7–M8 is made up with 20 particular regression dependencies. Consensus models M3, M6 and M9 include 100 regression equations. However, as the QNA and MNA descriptors have no direct physical meaning, the regression equations constructed on their basis are not explicitly displayed in the GUSAR2019 program. Only the QSAR models

satisfying the abovementioned condition have been further used for numerical predicting lgk_7 for the compounds of the external training set.

3.4. Assessment of the Range of the Applicability

To assess the applicability of models, GUSAR 2019 provides three different approaches based on similarity, leverage, and accuracy previously described in detail [54,88].

Similarity. Using the Pearson correlation coefficients for each compound, we calculated the distances toward its nearest neighbors in the training set in the space of independent variables obtained after SCR. The compound is considered in the range of the model's applicability if the average value of these three distances is lower or equal to 0.7.

Leverage. The calculation of leverage allows estimating the contribution of each molecule to its own predicted value [54,88]:

$$\text{Leverage} = x^T \left(\mathbf{X}^\mathrm{T}\mathbf{X}\right)^{-1} x,$$

where x is the vector of descriptors of the tested compound and **X** is the matrix made up with rows corresponding to the descriptors of all the molecules of the training set [54]. The compound is considered out of the applicability range if its leverage is larger than 99 % in the distribution of the leverage values of the training set.

Accuracy degree (AD). Here, the prediction of the applicability range for each compound is calculated based on the prediction error for the three most similar compounds in the test set relative to the training set as a whole [54,88]:

$$\text{AD}_{value} = \text{RMSE}_{3NN} / \text{RMSE}_{train}$$

In the present study, a threshold value of 1 was used for AD.

4. Results

Table S2. The validation parameters of the QSPR models estimated using the Xternal Validation Plus 1.2 program based on the experimental and predicted lgk_7 values of the compounds form internal training sets TR1 and TR2[1];

$\Delta lgk_{7(TR1)} = \Delta lgk_{7(TR2)} = 7.057$.

Comments	Prediction parameters	QSAR model used for predicting lgk_7					
		TR1			TR2		
		M1	M2	M3	M4	M5	M6
Classical Metrics (100% data)	R^2	0.9890	0.9872	0.9911	0.9887	0.9882	0.9916
	R^2_0	0.9846	0.9821	0.9866	0.9850	0.9827	0.9866
	$R^{2\prime}_0$	0.9822	0.9791	0.9845	0.9829	0.9796	0.9844
	$\overline{R^2_m}$	0.9154	0.9074	0.9174	0.9207	0.9058	0.9147
	ΔR^2_m	0.0161	0.0187	0.0140	0.0155	0.0182	0.0140
	CCC	0.9917	0.9903	0.9928	0.9920	0.9905	0.9927
Classical Metrics (after removing 5% data with high residuals)	R^2	0.9868	0.9849	0.9896	0.9887	0.9850	0.9925
	R^2_0	0.9845	0.9837	0.9876	0.9870	0.9839	0.9903
	$R^{2\prime}_0$	0.9236	0.9338	0.9317	0.9353	0.9366	0.9364
	$\overline{R^2_m}$	0.9384	0.9496	0.9454	0.9419	0.9532	0.9414
	ΔR^2_m	0.0141	0.0149	0.0113	0.0132	0.0146	0.0099
	CCC	0.9916	0.9912	0.9932	0.9932	0.9912	0.9947
Mean absolute error and standard deviation for test set (100% data)	RMSE	0.1360	0.1468	0.1269	0.1387	0.1499	0.1313

[1] [1] Where R^2, R^2_0, and R'^2 are determination coefficients calculated with and without taking into account the origin; $\overline{R^2_m}$ is the averaged determination coefficient of the regression function, calculated using values of determination coefficients on the ordinate axis (R^2_m) and using them on the abscissa (R'^2_m) respectively; ΔR^2_m is the difference between R^2_m and R'^2_m; CCC is the concordance correlation coefficient; MAE is the mean absolute error; SD is the standard deviation; ωNis the percentage of training sets TR1 and TR2, for which the prediction error is less than the interval proportional to 0.1, 0.15, 0.20, and 0.25 of Δlgk_7 of training sets TR1 (a) and TR2 (b).

	MAE	0.1008	0.1075	0.0920	0.1072	0.1075	0.0940
	SD	0.0916	0.1003	0.0878	0.0884	0.1051	0.0922
	MAE+3·SD	0.3755	0.4086	0.3554	0.3724	0.4227	0.3705
Mean absolute error and standard deviation for test set (after removing 5% data with high residuals)	RMSE	0.1090	0.1107	0.0975	0.1128	0.1098	0.0976
	MAE	0.0855	0.0894	0.0765	0.0924	0.0879	0.0773
	SD	0.0679	0.0656	0.0607	0.0650	0.0661	0.0599
	MAE+3·SD	0.2892	0.2862	0.2586	0.2873	0.2861	0.2570
Distribution of prediction errors (in %)	ωN in range 0.10×$\Delta lgk_{7\,(TR)}$	0.000[a]	0.000[a]	0.000[a]	0.000[b]	0.000[b]	0.000[b]
	ωN in range 0.15×$\Delta lgk_{7\,(TR)}$	0.000[a]	0.000[a]	0.000[a]	0.000[b]	0.000[b]	0.000[b]
	ωN in range 0.20×$\Delta lgk_{7\,(TR)}$	0.000[a]	0.000[a]	0.000[a]	0.000[b]	0.000[b]	0.000[b]
	ωN in range 0.25×$\Delta lgk_{7\,(TR)}$	0.000[a]	0.000[a]	0.000[a]	0.000[b]	0.000[b]	0.000[b]
Prediction quality	-	Good					
Systematic error presence	-	Absent					

Table S3. The validation parameters of the QSPR models estimated using the Xternal Validation Plus 1.2 program based on the experimental and predicted lgk_7 values of the compounds form test sets TS1 and TS2[2]; $\Delta lgk_{7(TR1)} = \Delta lgk_{7(TR2)} = 7.057$; $\Delta lgk_{7(TS1)} = 4.009$; $\Delta lgk_{7(TS2)} = 3.148$.

Comments	Prediction parameters	QSAR model used for predicting lgk_7								
		TS1						TS2		
		M1	M2	M3	M4	M5	M6	M4	M5	M6
Classical Metrics (100% data)	R^2	0.6142	0.5738	0.6361	0.5055	0.4646	0.5298	0.6882	0.6120	0.6648
	R^2_0	0.6125	0.5734	0.6327	0.5053	0.4623	0.5298	0.6882	0.5943	0.6627
	R'^2_0	0.3083	0.2252	0.3384	0.0680	0.0061	0.1243	0.5503	0.5196	0.5483
	Q^2_{F1}	0.8949	0.8826	0.8999	0.8652	0.8511	0.8709	0.9226	0.8989	0.9162
	Q^2_{F2}	0.6117	0.5664	0.6303	0.5020	0.4500	0.5233	0.6664	0.5642	0.6388
	$\overline{R^2_m}$	0.4396	0.3987	0.4486	0.3458	0.3047	0.3626	0.5421	0.4880	0.5441
	ΔR^2_m	0.2936	0.3092	0.2913	0.3184	0.2943	0.3182	0.2414	0.1475	0.2355

[2] Where R^2, R^2_0, and R'^2 are determination coefficients calculated with and without taking into account the origin; $\overline{R^2_m}$ is the averaged determination coefficient of the regression function, calculated using values of determination coefficients on the ordinate axis (R^2_m) and using them on the abscissa (R'^2_m) respectively; ΔR^2_m is the difference between R^2_m and R'^2_m; Q^2_{F1} and Q^2_{F2}, are determination coefficients calculated for the compounds of test sets TS1 and TS2 taking into account the average lgk_7 value of the compounds from training and test sets, respectively; CCC is the concordance correlation coefficient; MAE is the mean absolute error; SD is the standard deviation; ωNis the percentage of test sets TS1 and TS2, for which the prediction error is less than the interval proportional to 0.1, 0.15, 0.20, and 0.25 of Δlgk_7 of training sets TR1 (a) and TR2 (b).

	CCC	0.7511	0.7233	0.7632	0.6758	0.6483	0.6926	0.8086	0.7715	0.7993
Classical Metrics (after removing 5% data with high residuals)	R^2	0.8204	0.7364	0.7715	0.7696	0.7289	0.7807	0.7765	0.8125	0.8071
	R^2_0	0.8115	0.7342	0.7701	0.7621	0.7263	0.7741	0.7739	0.7936	0.8013
	R'^2_0	0.5555	0.4652	0.5346	0.4750	0.4466	0.5005	0.6304	0.7650	0.7064
	Q^2_{F1}	0.9538	0.9390	0.9525	0.9426	0.9367	0.9468	0.9567	0.9608	0.9621
	Q^2_{F2}	0.7966	0.7312	0.7627	0.7473	0.7212	0.7656	0.7679	0.7896	0.7969
	$\overline{R^2_m}$	0.6798	0.6010	0.6726	0.6191	0.5892	0.6332	0.6934	0.7434	0.7353
	ΔR^2_m	0.1673	0.2191	0.1803	0.2046	0.2249	0.1964	0.1316	0.0319	0.0653
	CCC	0.8763	0.8371	0.8600	0.8433	0.8293	0.8563	0.8775	0.8998	0.8970
Mean absolute error and standard deviation for test set (100% data)	RMSEP	0.5989	0.6328	0.5844	0.6782	0.7127	0.6635	0.5044	0.5765	0.5249
	MAE	0.4134	0.4273	0.3931	0.4482	0.4696	0.4318	0.3546	0.3591	0.3472
	SD	0.4422	0.4764	0.4413	0.5195	0.5472	0.5142	0.3680	0.4626	0.4039
	MAE+3·SD	1.7401	1.8566	1.7170	2.0066	2.1112	1.9744	1.4586	1.7471	1.5588
Mean absolute error and standard deviation for test set (after removing 5% data with high residuals)	RMSEP	0.4133	0.4750	0.4186	0.4606	0.4838	0.4436	0.3870	0.3685	0.3620
	MAE	0.3146	0.3309	0.2986	0.3296	0.3442	0.3129	0.2945	0.2719	0.2740
	SD	0.2740	0.3485	0.3000	0.3289	0.3476	0.3215	0.2580	0.2555	0.2431
	MAE+3·SD	1.1367	1.3763	1.1985	1.3164	1.3871	1.2773	1.0684	1.0383	1.0032
Distribution of prediction errors (in %)	ωN in range $0.10\times\Delta lgk_{7\ (TR)}$	12.0000[a]	20.0000[a]	20.0000[a]	20.0000[b]	24.0000[b]	16.0000[b]	15.0000[b]	15.0000[b]	15.0000[b]
	ωN in range $0.15\times\Delta lgk_{7\ (TR)}$	12.0000[a]	12.0000[a]	12.0000[a]	12.0000[b]	12.0000[b]	12.0000[b]	5.0000[b]	5.0000[b]	5.0000[b]
	ωN in range $0.20\times\Delta lgk_{7\ (TR)}$	4.0000[a]	4.0000[a]	4.0000[a]	8.0000[b]	8.0000[b]	8.0000[b]	5.0000[b]	5.0000[b]	5.0000[b]
	ωN in range $0.25\times\Delta lgk_{7\ (TR)}$	4.0000[a]	4.0000[a]	0.0000[a]	4.0000[b]	8.0000[b]	4.0000[b]	0.0000[b]	5.0000[b]	0.0000[b]
Prediction quality	-					Good				
Systematic error presence	-					Absent				

Table S4. Prediction of the lgk_7 values for the TR1 compounds using models M1-M3.*

Name	lgk_7^{obs}	M1		M2		M3	
		lgk_7^{pred}	$\|\Delta lgk_7\|$	lgk_7^{pred}	$\|\Delta lgk_7\|$	lgk_7^{pred}	$\|\Delta lgk_7\|$
2	5.130	5.153	0.023	5.067	0.063	5.113	0.017
3	5.155	5.108	0.047	5.102	0.054	5.133	0.022
4	5.164	5.093	0.071	5.104	0.060	5.105	0.059
5	4.477	4.595	0.118	4.572	0.095	4.582	0.105
6	5.193	5.062	0.131	5.038	0.155	5.053	0.140
7	5.114	5.151	0.037	4.981	0.133	5.064	0.050
8	5.114	5.100	0.014	5.012	0.102	5.082	0.032
9	4.477	4.321	0.156	4.279	0.198	4.311	0.166
10	4.415	4.396	0.019	4.375	0.040	4.400	0.015
12	5.230	5.128	0.102	5.021	0.209	5.081	0.149
14	4.477	4.582	0.105	4.596	0.119	4.587	0.110
15	5.050	5.051	0.001	5.020	0.030	5.045	0.005
16	5.160	5.128	0.032	5.076	0.084	5.112	0.048
17	5.130	5.083	0.048	5.044	0.086	5.061	0.069
18	5.000	5.071	0.071	5.102	0.102	5.076	0.076
19	5.000	4.814	0.186	4.889	0.111	4.847	0.153
20	5.040	5.051	0.011	5.010	0.030	5.023	0.017
22	4.380	4.481	0.101	4.583	0.203	4.496	0.116
23	4.580	4.534	0.046	4.450	0.130	4.491	0.089
24	5.274	5.218	0.056	5.117	0.157	5.189	0.085
26	4.040	4.047	0.007	4.078	0.038	4.061	0.021
27	4.380	4.538	0.158	4.482	0.102	4.509	0.129
28	4.340	4.539	0.199	4.446	0.106	4.487	0.147
29	5.170	5.160	0.010	5.156	0.014	5.162	0.008
30	4.800	4.769	0.031	4.771	0.029	4.793	0.007
31	5.176	5.157	0.019	5.097	0.080	5.139	0.037
32	5.120	5.023	0.097	5.011	0.109	5.032	0.088
33	4.660	4.688	0.028	4.742	0.082	4.696	0.036
35	5.200	5.217	0.017	5.199	0.001	5.194	0.006
36	5.114	5.064	0.050	5.067	0.047	5.026	0.088
37	3.568	3.527	0.041	3.728	0.160	3.645	0.077
38	4.680	4.508	0.172	4.421	0.259	4.460	0.220
39	3.810	3.889	0.079	3.916	0.106	3.882	0.072
41	4.610	4.592	0.019	4.522	0.088	4.570	0.040
42	5.200	5.229	0.029	5.210	0.010	5.206	0.006
44	4.544	4.646	0.102	4.528	0.016	4.592	0.048
45	3.756	3.863	0.107	3.934	0.178	3.912	0.156
47	4.205	4.105	0.100	4.120	0.085	4.136	0.069
48	4.280	4.174	0.106	4.296	0.016	4.254	0.026
49	3.670	3.753	0.083	3.740	0.070	3.769	0.099
50	3.755	4.128	0.373	3.853	0.098	3.939	0.184
51	4.090	4.045	0.045	3.983	0.107	4.025	0.065

52	4.220	4.068	0.152	4.152	0.068	4.141	0.079
53	4.200	4.185	0.015	4.277	0.077	4.234	0.034
54	4.300	4.360	0.059	4.348	0.048	4.348	0.048
55	4.335	4.421	0.086	4.273	0.062	4.350	0.015
56	4.320	4.477	0.157	4.329	0.009	4.396	0.076
58	4.640	4.541	0.099	4.551	0.089	4.551	0.089
59	4.560	4.524	0.036	4.530	0.030	4.528	0.032
60	4.675	4.683	0.008	4.642	0.034	4.662	0.013
61	4.755	4.931	0.176	4.668	0.087	4.786	0.031
63	4.130	4.214	0.084	4.268	0.138	4.247	0.117
64	4.155	4.204	0.049	4.245	0.090	4.214	0.058
65	3.245	3.493	0.248	3.543	0.298	3.531	0.286
66	4.200	4.298	0.098	4.225	0.024	4.268	0.067
67	4.560	4.557	0.003	4.457	0.103	4.511	0.049
68	4.200	4.451	0.251	4.329	0.129	4.415	0.215
69	3.830	3.928	0.098	3.961	0.131	3.945	0.115
70	3.230	3.508	0.278	3.489	0.259	3.471	0.241
72	4.205	4.258	0.053	4.258	0.053	4.254	0.049
73	4.470	4.699	0.229	4.518	0.048	4.563	0.093
74	4.930	5.074	0.144	4.891	0.039	4.974	0.044
76	4.820	4.659	0.161	4.615	0.205	4.646	0.174
77	4.480	4.460	0.020	4.507	0.027	4.460	0.020
78	5.283	5.230	0.054	5.276	0.007	5.246	0.037
79	5.297	5.241	0.056	5.311	0.014	5.282	0.015
80	5.348	5.374	0.026	5.356	0.008	5.363	0.015
81	5.260	5.120	0.140	5.189	0.071	5.160	0.100
82	5.297	5.284	0.013	5.350	0.053	5.312	0.015
83	5.130	5.147	0.017	5.005	0.125	5.079	0.051
84	5.330	5.399	0.069	5.377	0.047	5.387	0.057
85	0.000	0.330	0.330	0.455	0.455	0.348	0.348
86	0.000	0.412	0.412	0.585	0.585	0.454	0.454
87	6.462	6.370	0.093	6.370	0.092	6.366	0.097
88	6.114	6.122	0.008	6.131	0.017	6.137	0.023
89	6.146	6.156	0.010	6.107	0.040	6.154	0.008
90	5.643	5.776	0.132	5.805	0.162	5.806	0.163
91	6.732	6.550	0.183	6.606	0.126	6.607	0.125
92	6.756	6.643	0.113	6.623	0.133	6.638	0.118
93	6.204	6.318	0.114	6.265	0.061	6.272	0.068
94	6.431	6.298	0.133	6.358	0.074	6.347	0.085
95	6.431	6.424	0.008	6.408	0.024	6.397	0.034
96	6.176	6.163	0.013	6.138	0.038	6.152	0.024
98	6.255	6.200	0.055	6.216	0.040	6.199	0.057
99	6.279	6.263	0.016	6.285	0.006	6.269	0.010
100	6.431	6.279	0.153	6.305	0.126	6.304	0.127
101	6.580	6.492	0.088	6.510	0.070	6.528	0.052
104	5.944	5.932	0.013	5.987	0.042	5.974	0.029

105	6.041	5.888	0.153	5.991	0.051	5.958	0.083
106	6.279	6.026	0.253	6.132	0.146	6.122	0.157
108	6.176	6.124	0.052	6.147	0.029	6.133	0.043
109	6.322	6.234	0.089	6.258	0.065	6.238	0.084
110	6.041	6.003	0.038	6.068	0.026	6.028	0.014
111	6.000	6.029	0.029	6.072	0.072	6.035	0.035
113	6.322	6.304	0.018	6.277	0.046	6.304	0.019
114	6.223	6.204	0.019	6.206	0.017	6.200	0.023
115	6.255	6.161	0.094	6.234	0.021	6.214	0.041
117	5.933	5.912	0.021	5.996	0.063	5.948	0.015
118	7.057	6.773	0.284	6.772	0.285	6.800	0.257
121	5.114	5.407	0.293	5.400	0.286	5.388	0.274
122	6.279	6.175	0.104	6.143	0.136	6.168	0.111
123	5.954	5.960	0.006	6.000	0.045	5.977	0.023
124	6.204	6.156	0.049	6.192	0.012	6.172	0.032
125	6.301	6.158	0.143	6.147	0.154	6.136	0.165
126	6.079	6.206	0.127	5.989	0.091	6.051	0.028
127	6.505	6.427	0.078	6.428	0.077	6.425	0.080
128	6.176	6.018	0.158	6.020	0.156	6.002	0.174
129	6.204	5.916	0.289	5.908	0.296	5.942	0.263
130	6.176	6.091	0.085	5.980	0.196	6.024	0.152
131	4.531	4.442	0.090	4.726	0.195	4.607	0.076
132	5.544	5.469	0.076	5.611	0.066	5.540	0.004
133	5.491	5.583	0.091	5.269	0.222	5.438	0.053
134	6.230	6.117	0.113	6.167	0.064	6.129	0.101
135	5.771	5.867	0.096	5.869	0.098	5.853	0.082
137	3.301	3.706	0.405	3.717	0.416	3.700	0.399
139	5.204	5.185	0.019	5.183	0.021	5.177	0.027
141	4.602	4.945	0.343	4.960	0.357	4.914	0.312
142	5.114	5.241	0.127	5.365	0.251	5.319	0.205
143	5.000	5.146	0.146	5.181	0.181	5.201	0.201
144	3.431	3.766	0.335	3.882	0.450	3.803	0.372
145	4.398	4.486	0.088	4.475	0.077	4.482	0.084
146	4.398	4.405	0.007	4.417	0.019	4.422	0.024
147	4.699	4.607	0.092	4.584	0.115	4.606	0.093

* The falling out results are marked by red.

Table S5. Prediction of the lgk_7 values for the TR2 compounds using models M4-M6.*

Name	lgk_7^{obs}	M7		M8		M9	
		lgk_7^{pred}	$\lvert\Delta lgk_7\rvert$	lgk_7^{pred}	$\lvert\Delta lgk_7\rvert$	lgk_7^{pred}	$\lvert\Delta lgk_7\rvert$
2	5.130	5.168	0.038	5.008	0.122	5.099	0.031
3	5.155	5.091	0.064	5.078	0.077	5.120	0.035
4	5.164	5.081	0.083	5.039	0.125	5.084	0.080
5	4.477	4.577	0.100	4.572	0.095	4.572	0.095
6	5.193	5.039	0.154	5.029	0.164	5.041	0.153

7	5.114	5.156	0.042	4.959	0.155	5.057	0.057
8	5.114	5.096	0.018	5.053	0.061	5.079	0.035
9	4.477	4.269	0.209	4.219	0.258	4.268	0.209
10	4.415	4.408	0.007	4.362	0.053	4.401	0.014
12	5.230	5.103	0.127	5.008	0.222	5.071	0.159
15	5.050	5.089	0.039	5.066	0.016	5.071	0.021
16	5.160	5.162	0.002	5.157	0.003	5.136	0.024
17	5.130	5.046	0.084	5.076	0.054	5.060	0.070
19	5.000	4.921	0.079	4.946	0.054	4.929	0.071
20	5.040	5.015	0.026	5.021	0.019	5.014	0.026
22	4.380	4.476	0.096	4.557	0.177	4.490	0.110
24	5.274	5.231	0.043	5.100	0.175	5.178	0.096
26	4.040	4.078	0.038	4.080	0.040	4.073	0.033
27	4.380	4.523	0.143	4.493	0.113	4.515	0.135
28	4.340	4.597	0.257	4.471	0.131	4.520	0.180
29	5.170	5.163	0.007	5.142	0.028	5.156	0.014
30	4.800	4.749	0.051	4.742	0.058	4.777	0.023
32	5.120	5.001	0.119	4.945	0.175	5.002	0.118
33	4.660	4.648	0.012	4.709	0.049	4.658	0.002
35	5.200	5.200	0.000	5.149	0.051	5.164	0.036
36	5.114	5.012	0.102	5.002	0.112	5.000	0.114
37	3.568	3.466	0.102	3.698	0.130	3.619	0.051
39	3.810	3.894	0.084	3.845	0.035	3.877	0.067
41	4.610	4.645	0.034	4.477	0.133	4.565	0.045
42	5.200	5.189	0.011	5.190	0.010	5.195	0.005
44	4.544	4.663	0.119	4.533	0.011	4.607	0.063
45	3.756	3.828	0.072	3.873	0.117	3.873	0.117
47	4.205	4.107	0.098	4.109	0.096	4.123	0.082
48	4.280	4.189	0.091	4.262	0.018	4.248	0.032
49	3.670	3.753	0.083	3.768	0.098	3.763	0.093
50	3.755	3.955	0.200	3.784	0.029	3.863	0.108
51	4.090	4.024	0.066	3.991	0.099	4.032	0.058
52	4.220	4.051	0.169	4.171	0.050	4.131	0.090
53	4.200	4.202	0.002	4.253	0.053	4.237	0.037
55	4.335	4.428	0.093	4.205	0.130	4.346	0.011
56	4.320	4.485	0.165	4.374	0.054	4.412	0.091
58	4.640	4.556	0.084	4.564	0.076	4.565	0.075
59	4.560	4.550	0.010	4.547	0.013	4.553	0.007
60	4.675	4.681	0.006	4.632	0.043	4.672	0.003
61	4.755	4.916	0.161	4.675	0.080	4.776	0.021
63	4.130	4.233	0.103	4.325	0.195	4.273	0.143
64	4.155	4.241	0.086	4.267	0.111	4.253	0.098
65	3.245	3.515	0.270	3.513	0.268	3.536	0.291
67	4.560	4.635	0.075	4.495	0.065	4.563	0.003
68	4.200	4.453	0.253	4.346	0.146	4.404	0.204
70	3.230	3.486	0.256	3.538	0.308	3.508	0.278

72	4.205	4.261	0.056	4.249	0.044	4.271	0.066
73	4.470	4.705	0.235	4.511	0.041	4.571	0.101
74	4.930	5.093	0.163	4.920	0.010	4.972	0.042
76	4.820	4.669	0.151	4.651	0.169	4.639	0.181
77	4.480	4.457	0.023	4.513	0.033	4.464	0.016
78	5.283	5.239	0.044	5.256	0.027	5.238	0.045
79	5.297	5.265	0.032	5.327	0.030	5.292	0.005
82	5.297	5.311	0.014	5.352	0.055	5.316	0.019
84	5.330	5.417	0.087	5.366	0.036	5.384	0.053
85	0.000	0.146	0.146	0.499	0.499	0.272	0.272
86	0.000	0.422	0.422	0.611	0.611	0.504	0.504
87	6.462	6.367	0.096	6.387	0.076	6.372	0.090
89	6.146	6.130	0.016	6.135	0.011	6.137	0.009
90	5.643	5.780	0.137	5.797	0.154	5.812	0.169
91	6.732	6.533	0.200	6.574	0.158	6.584	0.149
92	6.756	6.628	0.128	6.590	0.166	6.623	0.133
94	6.431	6.296	0.135	6.314	0.118	6.317	0.115
95	6.431	6.375	0.056	6.402	0.030	6.387	0.045
96	6.176	6.154	0.022	6.182	0.006	6.153	0.023
98	6.255	6.232	0.023	6.205	0.051	6.222	0.033
99	6.279	6.308	0.029	6.300	0.021	6.283	0.004
100	6.431	6.309	0.123	6.330	0.101	6.304	0.128
105	6.041	5.918	0.124	5.994	0.047	5.951	0.090
106	6.279	6.012	0.266	6.099	0.180	6.107	0.172
108	6.176	6.111	0.065	6.135	0.041	6.132	0.044
109	6.322	6.227	0.095	6.269	0.053	6.238	0.084
110	6.041	6.011	0.030	6.068	0.027	6.035	0.006
111	6.000	6.049	0.049	6.091	0.090	6.049	0.049
114	6.223	6.050	0.173	6.170	0.053	6.120	0.103
117	5.933	5.803	0.130	5.984	0.051	5.904	0.029
118	7.057	6.750	0.307	6.739	0.318	6.778	0.279
122	6.279	6.258	0.020	6.286	0.008	6.264	0.015
123	5.954	6.001	0.047	6.047	0.093	6.012	0.057
124	6.204	6.153	0.051	6.173	0.032	6.167	0.037
125	6.301	6.162	0.139	6.250	0.051	6.209	0.092
126	6.079	6.248	0.169	5.948	0.131	6.017	0.062
127	6.505	6.405	0.100	6.439	0.066	6.425	0.080
128	6.176	6.136	0.040	6.118	0.059	6.115	0.062
129	6.204	5.900	0.304	5.949	0.255	5.955	0.249
130	6.176	6.087	0.089	6.003	0.173	6.020	0.156
131	4.531	4.459	0.072	4.679	0.147	4.568	0.037
132	5.544	5.433	0.111	5.543	0.001	5.520	0.024
133	5.491	5.586	0.095	5.283	0.208	5.431	0.060
134	6.230	6.102	0.129	6.140	0.091	6.117	0.113
135	5.771	5.862	0.092	5.857	0.086	5.851	0.080
137	3.301	3.755	0.454	3.747	0.446	3.801	0.500

139	5.204	5.392	0.188	5.250	0.046	5.262	0.058
141	4.602	4.925	0.323	4.962	0.360	4.932	0.330
142	5.114	5.259	0.145	5.393	0.279	5.319	0.205
143	5.000	5.134	0.134	5.165	0.165	5.175	0.175
145	4.398	4.521	0.123	4.483	0.085	4.557	0.159
147	4.699	4.710	0.011	4.720	0.021	4.759	0.060

* The falling out results are marked by red.

Table S6. Prediction of the lgk_7 values for the TS1 compounds using models M1-M3.*

Name	lgk_7^{obs}	M1		M2		M3	
		lgk_7^{pred}	$\lvert\Delta lgk_7\rvert$	lgk_7^{pred}	$\lvert\Delta lgk_7\rvert$	lgk_7^{pred}	$\lvert\Delta lgk_7\rvert$
1	4.520	4.649	0.129	4.769	0.249	4.734	0.214
11	5.240	4.858	0.382	4.566	0.674	4.709	0.531
13	4.680	4.217	0.463	4.334	0.346	4.330	0.350
21	4.415	4.308	0.107	4.787	0.372	4.476	0.061
25	5.137	5.167	0.030	5.023	0.114	5.099	0.038
34	5.114	5.410	0.296	5.177	0.063	5.201	0.087
40	4.280	3.959	0.321	4.343	0.063	4.154	0.126
43	5.180	5.184	0.004	5.016	0.164	5.123	0.057
46	4.360	4.663	0.303	5.162	0.802	4.807	0.446
57	4.590	4.642	0.052	4.638	0.048	4.670	0.080
62	4.200	4.138	0.062	4.219	0.019	4.214	0.014
71	3.000	4.206	1.206	4.287	1.287	4.229	1.229
75	5.310	4.804	0.506	4.254	1.056	4.437	0.873
97	6.041	6.273	0.231	6.269	0.227	6.248	0.207
102	6.322	5.833	0.489	6.099	0.223	5.970	0.353
103	6.204	5.572	0.633	5.938	0.266	5.839	0.366
107	5.663	5.542	0.121	5.791	0.128	5.775	0.112
112	6.255	6.179	0.076	6.257	0.002	6.204	0.052
116	6.158	5.974	0.185	6.124	0.035	6.064	0.095
119	7.009	6.320	0.688	6.311	0.698	6.300	0.709
120	6.447	6.034	0.414	6.044	0.403	6.127	0.320
136	4.000	5.893	1.893	5.765	1.765	5.731	1.731
138	3.602	4.808	1.206	4.909	1.307	4.819	1.217
140	5.041	5.272	0.231	5.083	0.041	5.245	0.203
148	4.875	4.568	0.307	4.545	0.330	4.518	0.357

* The falling out results are marked by red.

Table S7. Prediction of the lgk_7 values for the TS1 compounds using models M4-M6.*

Name	lgk_7^{obs}	M4		M5		M6	
		lgk_7^{pred}	$\lvert\Delta lgk_7\rvert$	lgk_7^{pred}	$\lvert\Delta lgk_7\rvert$	lgk_7^{pred}	$\lvert\Delta lgk_7\rvert$
1	4.520	4.613	0.093	4.748	0.228	4.712	0.192
11	5.240	5.035	0.205	4.507	0.733	4.713	0.527
13	4.680	4.250	0.430	4.321	0.359	4.322	0.358
21	4.415	4.295	0.120	4.774	0.359	4.512	0.097
25	5.137	5.201	0.064	5.002	0.135	5.108	0.029

34	5.114	5.363	0.249	5.044	0.070	5.155	0.041
40	4.280	4.075	0.205	4.216	0.065	4.092	0.188
43	5.180	5.221	0.041	4.990	0.190	5.110	0.070
46	4.360	4.362	0.002	5.028	0.668	4.679	0.319
57	4.590	4.677	0.087	4.631	0.040	4.643	0.053
62	4.200	4.191	0.009	4.284	0.084	4.256	0.056
71	3.000	4.284	1.284	4.286	1.286	4.295	1.295
75	5.310	4.637	0.674	4.267	1.043	4.332	0.978
97	6.041	6.419	0.377	6.323	0.281	6.331	0.290
102	6.322	5.737	0.585	6.070	0.252	5.934	0.388
103	6.204	5.380	0.825	5.790	0.414	5.755	0.449
107	5.663	5.682	0.019	5.832	0.169	5.808	0.145
112	6.255	6.172	0.083	6.228	0.028	6.192	0.063
116	6.158	5.853	0.306	6.227	0.068	6.024	0.134
119	7.009	6.265	0.744	6.175	0.834	6.318	0.691
120	6.447	5.823	0.624	5.978	0.470	5.941	0.507
136	4.000	5.977	1.977	5.904	1.904	5.858	1.858
138	3.602	5.249	1.647	5.524	1.922	5.342	1.740
140	5.041	5.470	0.429	5.147	0.105	5.313	0.271
148	4.875	4.749	0.126	4.840	0.035	4.820	0.055

* The falling out results are marked by red.

Table S8. Prediction of the lgk_7 values for the TS2 compounds using models M4-M6.*

Name	lgk_7 ^{obs}	M4		M5		M6	
		lgk_7 ^{pred}	$\|\Delta lgk_7\|$	lgk_7 ^{pred}	$\|\Delta lgk_7\|$	lgk_7 ^{pred}	$\|\Delta lgk_7\|$
14	4.477	4.863	0.386	4.847	0.370	4.828	0.351
18	5.000	5.176	0.176	5.306	0.306	5.223	0.223
23	4.580	4.657	0.077	4.213	0.367	4.408	0.172
31	5.176	5.138	0.038	5.042	0.134	5.102	0.074
38	4.680	3.910	0.770	3.809	0.871	3.803	0.877
54	4.300	4.383	0.083	4.299	0.002	4.396	0.096
66	4.200	4.562	0.362	4.236	0.036	4.413	0.213
69	3.830	4.207	0.377	4.149	0.319	4.217	0.387
80	5.348	5.370	0.022	5.296	0.052	5.322	0.026
81	5.260	4.893	0.368	5.070	0.190	4.976	0.284
83	5.130	5.074	0.056	4.858	0.272	4.985	0.145
88	6.114	6.114	0.000	6.249	0.135	6.160	0.046
93	6.204	6.577	0.373	6.379	0.175	6.410	0.206
101	6.580	6.373	0.207	6.495	0.085	6.480	0.100
104	5.944	6.073	0.128	6.068	0.123	6.064	0.119
113	6.322	6.126	0.196	6.073	0.250	6.083	0.239
115	6.255	5.622	0.633	6.201	0.054	5.981	0.275
121	5.114	6.016	0.902	6.012	0.898	5.977	0.863
144	3.431	4.929	1.498	5.448	2.017	5.169	1.738
146	4.398	4.841	0.443	4.927	0.529	4.909	0.511

* The falling out results are marked by red.
